# Targeted Downregulation of s36 Protein Unearths its Cardinal Role in Chorion Biogenesis and Architecture during *Drosophila melanogaster* Oogenesis

**DOI:** 10.1038/srep35511

**Published:** 2016-10-18

**Authors:** Athanassios D. Velentzas, Panagiotis D. Velentzas, Niki E. Sagioglou, Eumorphia G. Konstantakou, Athanasios K. Anagnostopoulos, Maria M. Tsioka, Vassiliki E. Mpakou, Zoe Kollia, Christos Consoulas, Lukas H. Margaritis, Issidora S. Papassideri, George Th. Tsangaris, Evangelia Sarantopoulou, Alkiviadis-Constantinos Cefalas, Dimitrios J. Stravopodis

**Affiliations:** 1 Section of Cell Biology and Biophysics, Department of Biology, School of Science, National and Kapodistrian University of Athens (NKUA), Athens, Greece; 2 Proteomics Core Facility, Systems Biology Center, Biomedical Research Foundation of the Academy of Athens (BRFAA), Athens, Greece; 3 Theoretical and Physical Chemistry Institute, National Hellenic Research Foundation (NHRF), Athens, Greece; 4 Laboratory of Experimental Physiology, Medical School, National and Kapodistrian University of Athens (NKUA), Athens, Greece

## Abstract

*Drosophila* chorion represents a model biological system for the *in vivo* study of gene activity, epithelial development, extracellular-matrix assembly and morphogenetic-patterning control. It is produced during the late stages of oogenesis by epithelial follicle cells and develops into a highly organized multi-layered structure that exhibits regional specialization and radial complexity. Among the six major proteins involved in chorion’s formation, the s36 and s38 ones are synthesized first and regulated in a cell type-specific and developmental stage-dependent manner. In our study, an RNAi-mediated silencing of *s36* chorionic-gene expression specifically in the follicle-cell compartment of *Drosophila* ovary unearths the essential, and far from redundant, role of s36 protein in patterning establishment of chorion’s regional specialization and radial complexity. Without perturbing the developmental courses of follicle- and nurse-cell clusters, the absence of s36 not only promotes chorion’s fragility but also induces severe structural irregularities on chorion’s surface and entirely impairs fly’s fertility. Moreover, we herein unveil a novel function of s36 chorionic protein in the regulation of number and morphogenetic integrity of dorsal appendages in follicles sporadically undergoing aged fly-dependent stress.

*Drosophila* oogenesis represents a fundamental developmental process and an attractive biological platform for the analysis of gene regulation, cell differentiation and tissue morphogenesis^1^. The egg chamber, or otherwise follicle, constitutes the structural and functional unit of fly ovary and is formed in the germarium at the tip of the ovary^1^,^2^. Each follicle consists of 16 germ-line cells (15 nurse cells and 1 oocyte) surrounded by a monolayer of approximately 650 somatic epithelial cells called follicle cells^3^. The development of a typical follicle is divided into several discrete stages (14 according to King^2^ and 20 according to Margaritis^4^,^5^) based on anatomical and morphological criteria. These include follicle size, relative proportion of follicle’s volume occupied by the oocyte, eggshell formation and appearance of specialized structures.

*Drosophila* eggshell is produced during the late stages of oogenesis by epithelial follicle cells and is assembled as a highly organized multi-layered structure that exhibits regional and radial complexity^4^,^6^,^7^. Its radial complexity is recognized in the main-body region where five distinct and successive layers can be morphologically identified. These are: an oocyte proximal vitelline membrane (~300 nm), a lipid wax layer, an inner chorionic layer or ICL (~40–50 nm), a tripartite endochorion that consists of floor, pillars and roof (~500–700 nm), and an outermost non-proteinaceous exochorion (~300–500 nm). The ICL, endochorion and exochorion are collectively termed as the chorion^4^,^6^,^7^,^8^. Six major proteins produced by epithelial follicle cells have been detected in *Drosophila* chorion. Their sequential synthesis and secretion require precise regulation of gene-amplification and gene-expression mechanisms^9^,^10^,^11^. Based on their developmentally distinct patterns of expression, they are categorized as “early” (s36 and s38; stages 11–13), “middle” (s16 and s19; stage 13) and “late” (s15 and s18; stage 14) chorionic proteins^7^,^8^ with s36 and s38 having been thought to play a major role in early choriogenesis.

Each follicle is presented with regionally specialized structures formed either at the anterior end, such as the micropyle, operculum, collar and dorsal appendages, or at the posterior pole, such as the aeropyle. Micropyle is a narrow channel through which sperm enters into the oocyte, while operculum is a weakened region of the eggshell that facilitates hatching of the larva at the end of embryogenesis^4^,^7^. Dorsal appendages are long (~300 μm) cylindrical structures with paddle-shaped tips located on either side of the midline. They largely consist of a modified pillars’ network with air-spaces among them that allow eggs to facilitate gas exchange. Appendages’ synthesis begins at stage 11 by engagement of approximately 65–70 follicle cells^1^,^4^,^7^,^12^. At the end of oogenesis, during oviposition, the follicle cells slough off and leave characteristic pentagonal and hexagonal imprints on the external surface of laid eggs.

Hence, in an effort to reliably assess the *in vivo* role of s36 protein during ovarian-chorion biogenesis, we have herein targeted the *s36* gene via an RNAi-based gene-silencing approach. Obtained data unveiled the essential contribution of s36 protein in the assembly and architecture of *Drosophila* chorion. s36 downregulation resulted in chorion’s disintegration and fly’s infertility. An unexpected age-dependent function of s36 as regulator of the number and morphogenetic patterning of dorsal appendages during choriogenesis was also unearthed.

## Materials and Methods

### *Drosophila melanogaster* strain stocks and culturing conditions

The following *D. melanogaster* transgenic fly strains were used: P{w[+mW.hs]=GawB}c355, w[1118] (BL: 3750), w[*]; P{w[+mW.hs]=GawB}109–28 (BL: 7022), P{w[+mW.hs]=GawB}109C1, y[1] w[*] (BL: 7020), w[1118]; P{w[+mW.hs]=GawB}shep[c522] (BL: 3747), y[*] w[*]; P{w[+mC]=UAS-2xEGFP} AH2 (BL: 6874), all obtained from Bloomington *Drosophila* Stock Center (Indiana, USA), and UAS-s36_RNAi (Transformant ID: 14824), provided by Vienna *Drosophila* RNAi Center (Vienna, Austria). Fly stocks were maintained at 25 °C and fed on standard diet (6.4% rice flour, 5% tomato paste, 3.2% sugar, 0.8% yeast, 0.8% agar, 0.4% ethanol and 0.4% propionic acid).

### RT-sqPCR

Total RNA from transgenic (control and s36-targeted) fly ovaries was extracted following a Trizol-based protocol (Molecular Research Center Inc., Ohio, USA). One microgram of RNA was reverse transcribed using an oligo-[dT]_12–18_ primer and the MMLV reverse transcriptase (Thermo Fisher Scientific Inc.-Life Technologies-Invitrogen, Massachusetts, USA). Produced cDNA was amplified by semi-quantitative (sq) PCR with a Bio-Rad T100 Thermocycler (Bio-Rad, California, USA), using chorionic gene-specific oligonucleotide primers (Table S1). PCR fragments were resolved in 2–3% agarose gels according to standard procedures. Data were collected from three independent experiments of different fly crosses per biological condition tested.

### Western blotting

Whole-ovary total-protein lysates derived from transgenic *D. melanogaster* female flies were prepared as previously described^13^. Briefly, equal amounts of approximately 50 μg proteins per experimental condition were analyzed by SDS-PAGE in 12% gels under reducing conditions (in the presence of 0.75 M β-mercaptoethanol). Separated proteins were electro-transferred onto nitrocellulose membranes with a buffer containing 40 mM glycine, 50 mM Tris-base, 0.04% SDS and 20% methanol following standard immunoblotting procedures, and successively probed with an s36-chorionic-protein rabbit antiserum (diluted 1:1000; kindly provided by Dr. Gail L. Waring, Department of Biology, Marquette University, Wisconsin, USA). Non-specific -polyclonal- antibody binding was eliminated by incubation of the nitrocellulose membranes with blocking solution (1x TBS, 0.1% Tween-20 and 5% NFM) for 2 h at room temperature. Following incubation with an HRP-conjugated secondary anti-rabbit antibody (Santa Cruz Biotechnology Inc., Texas, USA), the immunoreactive protein bands were visualized using an enhanced chemiluminescence (ECL) reagent kit (Perkin Elmer, Massachusetts, USA). Equal loading of each lane was evaluated by immunoblotting of the same membranes with an actin mouse monoclonal antibody (sc-81178, diluted 1:1000; Santa Cruz Biotechnology Inc., Texas, USA). Western blotting assays were repeated three times, using independent genetic crosses per experimental condition analyzed.

### Proteοmics: peptide generation – liquid chromatography-mass spectrometry/mass spectrometry (LC-MS/MS)

Total-protein samples were prepared from 30 s36-depleted (c355 > s36_RNAi) fly ovaries. Protein purification and processing for tryptic-peptide generation and extraction were carried out as previously described^14^. Produced peptides were analyzed using an LTQ Orbitrap Elite instrument (Thermo Scientific, Illinois, USA) with the mass spectrometer being coupled to a Dionex Ultimate 3000 HPLC system. For peptide separation, a C18 Acclaim Pepmap column of 15 cm was used and properly paired with an Acclaim Pepmap Nano-trap of 2 cm (Thermo Scientific, Illinois, USA). The extracted ion chromatogram was further processed using the Proteome Discoverer software (Thermo Scientific, Illinois, USA) and the Sequest search engine. The database chosen for protein identification searches was the *D. melanogaster* reference proteome from UniProt 2.16. Identification criteria included a precursor-mass tolerance of 10 ppm and fragment-mass tolerance of 0.05 Da. Trypsin was selected as the cleavage enzyme with a maximum of “0” missed-cleavage parameter. A false-discovery rate-threshold of 0.5% ensured the reliability of the herein described protein identifications. The proteomic content of s36-depleted ovary was thoroughly profiled via three independent fly crosses.

### Light microscopy (LM)

Collected *D. melanogaster* laid -transgenic- follicles were stained with 0.06% 3-3′-diaminobenzidine (DAB) in 50 mM Tris-HCl, pH 7.6 and examined under visible light with a BMS stereomicroscope. Propidium iodide (PI) staining of follicles and embryos was carried out as previously described^15^,^16^. Transgenic (s36-targeted) follicles stained with PI or over-expressing the eGFP fluorescent protein were visualized under a Nikon confocal laser scanning microscope (CLSM), model Digital Eclipse C1 (Nikon, Tokyo, Japan). For the neutral-red staining assay, de-chorionated follicles were incubated for 10 min at room temperature with a solution of 5 mg/ml neutral red (Sigma-Aldrich, Missouri, USA) in 1x PBS, subsequently washed five times with 1x PBS containing 0.05% Triton X-100 and finally visualized under a BMS stereomicroscope. LM imaging was repeated three times with independent fly crosses for each experimental condition studied.

### Scanning electron microscopy (SEM)

Surface structural organization and architecture of laid transgenic follicles from the different genetic-background strains examined herein were visualized through employment of SEM technology. Preparation for SEM analysis was performed as following: laid follicles were pre-fixed for 2 h at 4 °C in 1x PBS containing 2.5% glutaraldehyde, pH 7.4. Next, specimens were post-fixed in 2% osmium tetroxide for 1 h at 4 °C. Tissue dehydration was successively attained via a graded ethanol series (30%, 50%, 70%, 80%, 90%, 95% and 100%). Samples were, then, subjected to a critical-point drying (Samdri 780A; Tousimis, Maryland, USA) process, attached on aluminum stubs, coated with gold-palladium (60:40) in a sputter-coating apparatus (Samsputter 2a; Tousimis, Maryland, USA) and finally visualized under a Philips 515 scanning electron microscope. SEM imaging experiments were performed three times with independent genetic crosses for each examined condition.

### Transmission electron microscopy (TEM)

*D. melanogaster* follicles were processed for TEM as following: transgenic ovaries were carefully dissected, manually separated into individual follicles in Ringer’s solution and immediately fixed with 1x PBS containing 2% formaldehyde and 2% glutaraldehyde for 90 min at room temperature. Next, follicles were post-fixed for 60 min at 4 °C with 1x PBS containing 2% osmium tetroxide. Specimens were, then, gradually dehydrated in 30%, 50% and 70% ethanol for 10 min each, stained *en block* for 30 min in 70% ethanol containing 0.5% uranyl acetate, dehydrated in 80%, 90%, 95% and 100% ethanol for 10 min each, infiltrated in propylene oxide and finally embedded in Epon-Araldite epoxy resin (Fullam Inc., New York, USA). Ultrathin sections were mounted on uncoated copper grids, stained with uranyl acetate and lead citrate, and viewed under a Philips EM300 transmission electron microscope. TEM imaging experiments were repeated three different times, collecting data from independent fly crosses for each analyzed condition.

### Atomic force microscopy (AFM)

For a closer to an atomic resolution mapping of the morphological landscape of *D. melanogaster* ovarian-follicle eggshell surface and its physical properties, an atomic force microscope (di Innova; Bruker, California, USA) with a few nanometers resolution was engaged. AFM imaging and surface analysis were carried out in tapping mode, using for control follicles a 40 nN/nm stiff silicon cantilever (MPP-11123-10; Bruker, California, USA) at 300 kHz and for s36-downregulated follicles a 0.05 nN/nm soft silicon nitride MLCT cantilever at 38 kHz (“E” tip). Spring constants were measured via the thermal noise method. High-resolution images were acquired at a scanning rate of 0.2 Hz. Image processing and data acquisition were performed using the SPMLab v7.0 analysis software. The influence of s36 downregulation on the mechanical properties of *Drosophila* ovarian eggshell was determined via force-distance curves (F-D, indentation versus loading force). Mechanical strength of control and s36-depleted follicles was evaluated by locally applying an AFM-tip-compelled force and indenting eggshell’s surface. F-D curves of both ovarian-chorion types (control versus s36-targeted) analyzed herein were recorded on specific points regularly distributed over a selected area. The stiffness variation of s36-downregulated follicles was identified via elastic (Young’s) modulus, using stiff and soft cantilevers for control and s36-depleted chorion bodies, respectively. Positions close to the center of each examined follicle were suitably chosen to avoid large stiffness variations near the edges. Young’s modulus values were obtained by fitting the contact-region data of the retracted F-D curve to the Hertz-Sneddon model^17^ with a hysteresis correction of force-curve pair. AFM imaging experiments were carried out three times, collecting data from independent genetic crosses for both control and s36-downregulated biological settings.

### Hatchability assay

Five pairs of 3-day-old adult flies were transferred into fresh medium and remained there for eight hours to mate and lay their eggs. Hatching efficiency was quantified by comparing the number of laid eggs (as measured by a stereomicroscope) with the number of 3^rd^ instar larvae developed. Experiments were performed three different times, using independent genetic crosses for each biological condition examined.

## Results

### RNAi-mediated targeting of *s36* chorionic-gene expression in the follicle-cell compartment produces s36-depleted ovaries in *Drosophila melanogaster*

**Figure 1:**
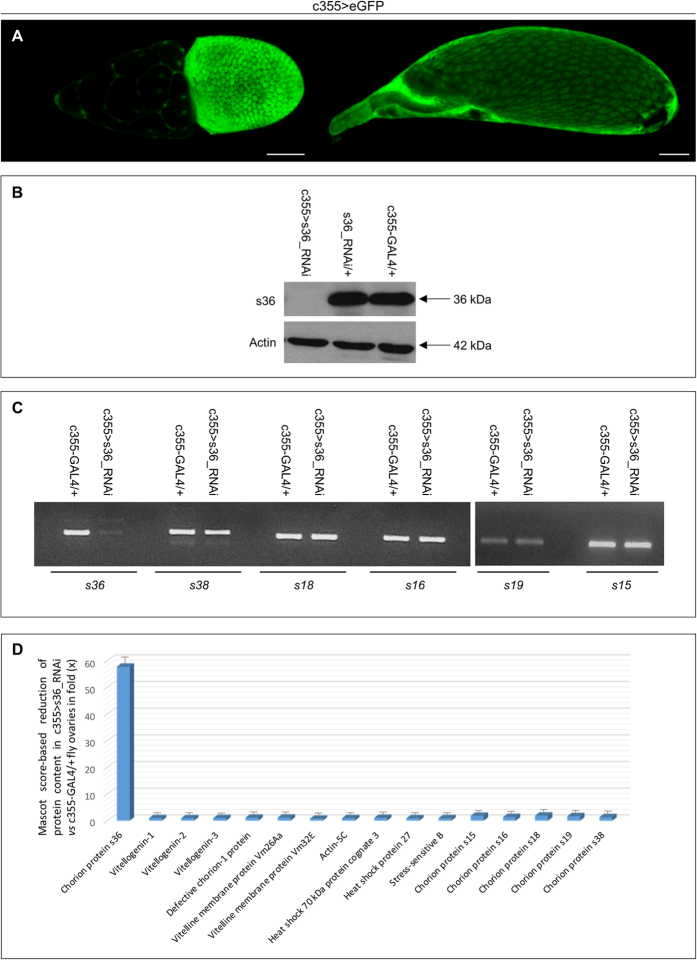
Follicle cell-specific and RNAi-mediated targeting of *s36* chorionic gene causes generation of s36 protein-depleted ovaries in *Drosophila melanogaster*. (**A**) Confocal laser scanning microscopy images of representative stage-10 (left) and late stage-13 (right) follicles of c355 > eGFP transgenic control cross. (**B**) Western blotting analysis of ovaries derived from s36-targeted (c355 > s36_RNAi) and control (c355-GAL4/+ and s36_RNAi/+) flies via utilization of an s36-specific antiserum. Actin served as protein of reference. (**C**) Gene-expression profiling of the six major chorionic genes *s36*, *s38*, *s18*, *s16*, *s19* and *s15* in s36-depleted (c355 > s36_RNAi) and control (c355-GAL4/+) ovaries through employment of an RT-sqPCR protocol (also, see Table S1). (**D**) Graphical presentation of comparative measurements (in fold: “x”) of relative protein abundance, using a Mascot score-based evaluation process, in between s36-targeted (c355 > s36_RNAi) (also, see Table S2) and control (c355-GAL4/+)^14^ ovaries. Scale Bars: 50 μm.

To investigate the *in vivo* role of s36 chorionic protein during the course of *Drosophila* eggshell development, transgenic flies were generated through employment of the GAL4/UAS binary genetic system, directing the RNAi-induced silencing of *s36* chorionic gene specifically in the follicle-cell compartment of fly ovary. Control-cross offspring carrying the herein chosen genetic driver (c355-GAL4) and the eGFP reporter protein revealed the follicle cell-specific pattern of driver’s promoter activity throughout late oogenesis (Fig. 1A and data not shown). Ovaries containing s36-targeted follicles (c355 > s36_RNAi) were subjected to Western blotting analysis via utilization of an s36-specific antiserum^18^ and proved to be depleted of the s36 chorionic protein as compared to control ovaries from both c355-GAL4/+ and s36_RNAi/+ flies (Fig. 1B). This demonstrates the successful disruption of *s36* gene expression despite its strong activity derived from a developmentally regulated gene-amplification of approximately 15 fold^10^,^11^. Moreover, it indicates the critical contribution of follicle-cell compartment to s36-protein synthesis and secretion in fly ovary.

Next, we tried to elucidate if the lack of s36 chorionic protein could affect the developmental courses of follicle- and nurse-cell compartments during *Drosophila melanogaster* oogenesis. Therefore, the eGFP protein was specifically expressed in s36-depleted follicle cells (Fig. S1A–D), while PI was used to stain the nurse-cell cluster of s36-targeted follicles (Fig. S1E–H). GFP expression revealed that in the absence of s36 protein the distribution of follicle cells remained unaltered throughout all stages of follicle development (Fig. S1A–D). Moreover, GFP synthesis suggested the transcriptional and translational integrity of follicle cells. In addition, PI-positive staining of s36-targeted follicles indicated the unaffected integrity of nurse-cell cluster (Fig. S1E–H).

**Figure 2:**
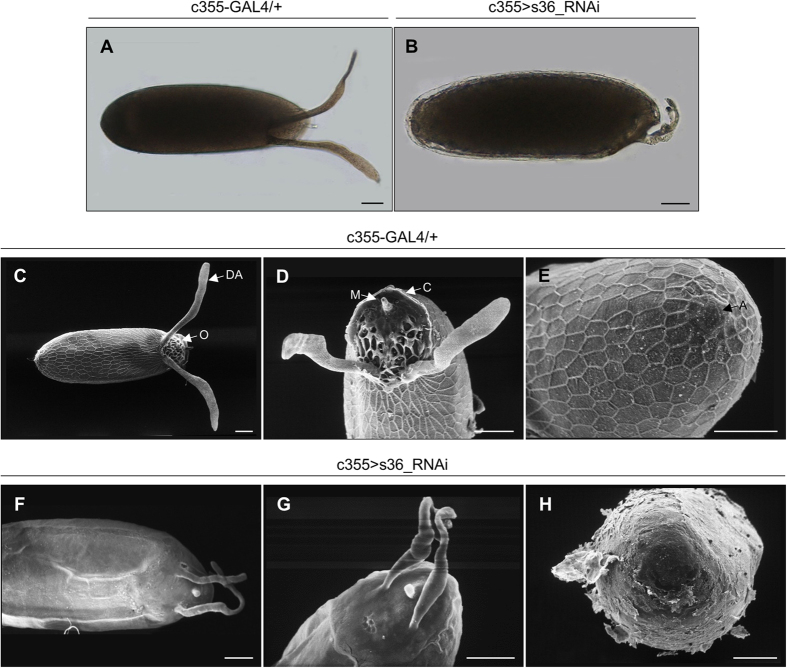
The s36 chorionic protein controls the development of regionally specialized structures of *Drosophila melanogaster* eggshell. (**A**,**B**) Light microscopy images of representative control (c355-GAL4/+) (**A**) and s36-targeted (c355 > s36_RNAi) (**B**) follicles. (**C–H**) Scanning electron microscopy images of representative control (**C**–**E**) and s36-depleted (**F**–**H**) follicles. (**C**,**F**) Far-off view of follicle’s general morphology. (**D**,**G**) Follicle’s anterior-end architecture. (**E**,**H**) Follicle’s posterior-end structure. DA: Dorsal Appendages, O: Operculum, M: Micropyle, C: Collar and A: Aeropyle. Scale Bars: 50 μm.

**Figure 3:**
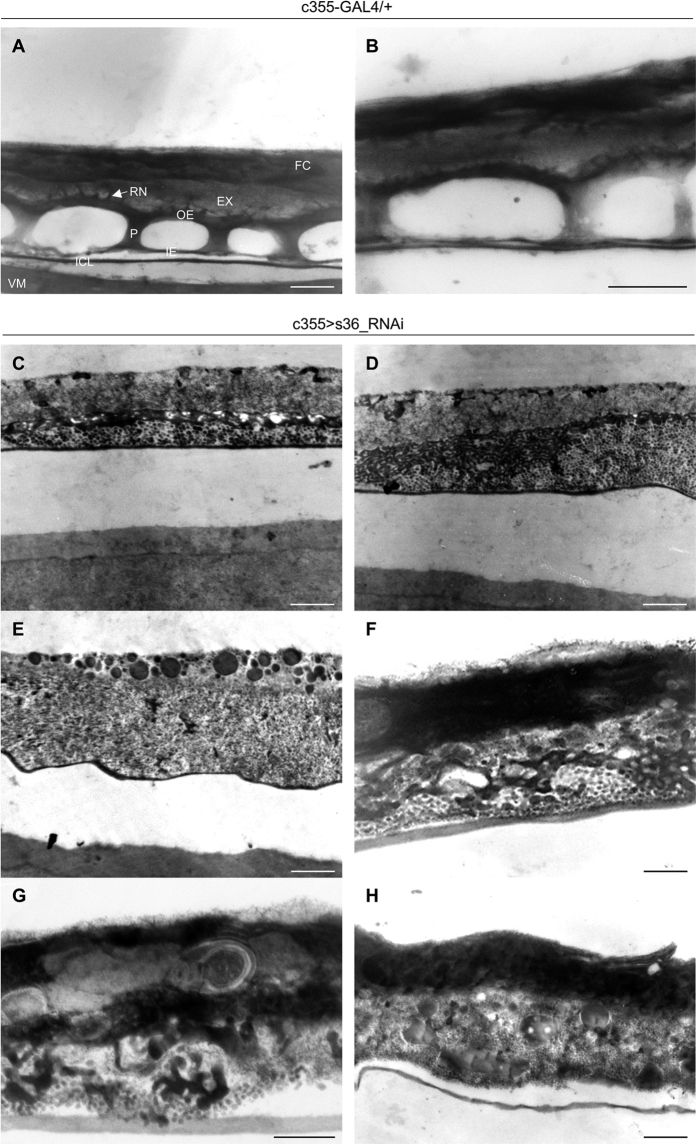
The indispensable contribution of s36 protein in biogenesis of chorion’s radial complexity. (**A**,**B**) Transmission electron micrographs of representative control (c355-GAL4/+) follicles. (**C**–**H**) Transmission electron microscopy images of eggshell’s ultrastructural organization in s36-downregulated (c355 > s36_RNAi) follicles. Phenotypic variation (**C**–**H**) dictates the ability of s36 to modulate developmental randomness likely evolved during chorion’s assembly. VM: Vitelline Membrane, ICL: Inner Chorionic Layer, IE: Inner Endochorion (Floor), P: Pillar(s), OE: Outer Endochorion (Roof), EX: Exochorion, RN: Roof Network and FC: Follicle Cell(s). Scale Bars: 0.5 μm.

To evaluate the targeting specificity of our gene-silencing strategy, we examined the transcription-activity profiling of the six major chorionic genes in control (c355-GAL4/+) and s36-depleted (c355 > s36_RNAi) ovaries via employment of an RT-sqPCR protocol. Interestingly, the *s36* gene activity proved to be significantly reduced in the *s36*-targeted ovaries as compared to control ones. In contrast, the other chorionic genes *s38*, *s18*, *s16*, *s19* and *s15* were presented with unharmed transcriptional expression (Fig. 1C). Therefore, downregulation of *s36* does not seem to affect the amplification and expression profiling of the other chorion-gene members. Furthermore, and due to the lack (except s36; Fig. 1B) of chorionic component-specific antibodies, the proteomic landscape of s36-depleted ovaries (c355 > s36_RNAi) was mapped and compared to *Drosophila* control (c355-GAL4/+) one recently reported^14^. LC-MS/MS analysis and processing of the generated unique peptides (17,772) through the *D. melanogaster* reference proteome resulted in the identification of 2,131 distinct proteins (Table S2). Comparative measurements (in fold: “x”) of protein abundance, using Mascot-score value as the evaluation criterion^19^, between the s36-targeted (c355 > s36_RNAi) (Table S2) and control (c355-GAL4/+)^14^ ovaries are graphically presented in Fig. 1D. Interestingly, over a 50x reduction of s36 protein content in s36-targeted versus control ovaries was observed. In contrast, a plethora of proteins critically implicated in vitellogenesis, choriogenesis and diverse structural (e.g. Actin-5C) or regulatory (e.g. Heat shock 70 kDa protein cognate 3) networks remained either unaffected (e.g. Vitellogenin-1) or were subjected to small drops in their total levels. Intriguingly, the five chorionic proteins s15, s16, s18, s19 and s38 were presented with slightly reduced contents in the s36-depleted versus control ovaries (Fig. 1D). Severe downregulation of s36 may prohibit the organized recruitment of other chorionic components to the likely defective chorionic structural scaffold (see Figs 2 and 3), therefore resulting in their inadequate representation in the final proteomic extracts examined.

### The indispensable role of s36 chorionic protein in morphogenesis of regionally specialized structures of *Drosophila melanogaster* eggshell

Follicles from s36-downregulated ovaries were presented with morphological deformities and anatomical defects, as revealed via thorough microscopic analysis. LM imaging of laid s36-targeted follicles were characterized by thin chorion and highly dysmorphic dorsal appendages (Fig. 2B) as compared to control follicles of the same developmental stage (Fig. 2A). SEM imaging of laid eggs (Fig. 2C–H) unearthed the disastrous impact of s36-protein lack on chorionic regional integrity. Specifically, in contrast to control follicles (Fig. 2C–E), s36-depleted ones were shown to carry abnormally thin and fragile eggshell (Fig. 2H), without follicular imprints, and with two considerably reduced in both length and robustness coiled dorsal appendages that also lacked their characteristic paddle-shaped tips (Fig. 2F,G). Follicles depleted of s36 were also deprived of the typical appearance of other specialized structures formed at both the anterior and posterior ends, such as the operculum, collar and aeropyle (Fig. 2G,H) as compared to control follicles (Fig. 2D,E). Micropyle, a critical follicle protrusion that plays an essential role for sperm entry in laid eggs (Fig. 2C,D), was also presented with serious deformities (e.g. small size and closed hole) in the vast majority of s36-downregulated follicles examined (Fig. 2F,G). These data demonstrate that s36 protein is required for proper chorion deposition and successful acquisition of its regionally specialized architecture.

### Loss of eggshell’s radial complexity in s36-depleted fly ovaries

Through employment of TEM technology and visualization of ultrathin cross-section images derived from stage 14 control follicles all the typical main-body eggshell structures could be clearly identified (Fig. 3A,B). The mature eggshell of *D. melanogaster* consists of a homogeneous, electron-dense, vitelline membrane (VM) adjacent to the oocyte (Fig. 3A), an inner chorionic layer (ICL), a tripartite endochorion (composed of the inner endochorion (IE) or floor, the pillars (P) and the outer endochorion (OE) or roof) and an outermost non-proteinaceous exochorion (EX). Roof is anchored into the exochorion with characteristic protrusions forming the roof network (RN) (Fig. 3A,B).

TEM-mediated ultrastructural analysis of s36-downregulated follicles provided solid proof for the essential contribution of s36 protein to the acquisition of eggshell’s radial complexity (Fig. 3C–H). Ultrathin sections of early-stage 14 follicles revealed a complete failure of endochorionic organization. The pathological phenotypes obtained, although they share in common the collapse of tripartite endochorion, vary considerably in appearance and strength (Fig. 3C–H). As illustrated in Fig. 3C,D, the floor and pillars are replaced by small clumps of endochorionic material, while a very thin roof can be identified. On the other hand, exochorion structure remains relatively unaffected, although a degree of morphological heterogeneity can be observed. Exochorion is assembled at developmental stage 14 during which chorionic genes from the 3^rd^-chromosome cluster remain highly active^8^,^20^. This suggests that the X chromosome-specific *s36* gene product is not directly involved in exochorion formation. In Fig. 3E–H, the roof can hardly be distinguished, while the exochorion layer fails to be properly anchored to a solid scaffold and collapses within the disintegrated endochorion. As shown in Fig. 3E, s36 downregulation is associated with accumulation of diverse-size and electron-dense vesicles, likely indicating the failure of follicle cells to incorporate necessary structural material into the developing eggshell.

**Figure 4:**
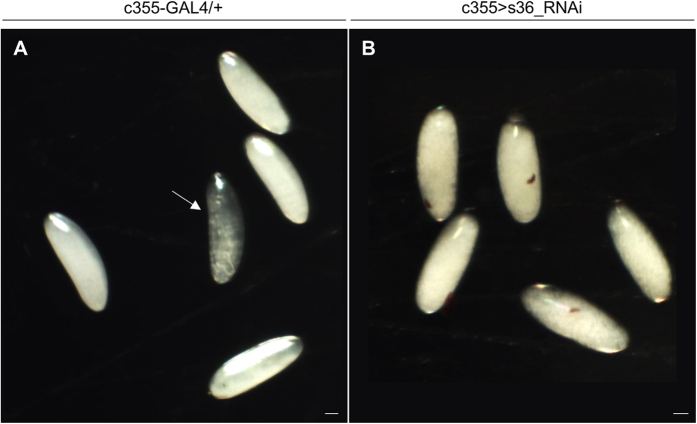
Targeted downregulation of s36 chorionic protein in the follicle-cell cluster of *Drosophila melanogaster* ovary does not seem to affect vitelline-membrane’s impermeability. Light microscopy images of control (c355-GAL4/+) (**A**) and s36-downregulated (c355 > s36_RNAi) (**B**) laid de-chorionated follicles after their incubation with neutral red, demonstrating the impermeability of control and s36-targeted follicles to the dye. The arrow in (**A**) indicates a developing embryo. Scale Bars: 50 μm.

In contrast to endochorion, ICL structural integrity does not seem to be affected in the s36-depleted follicles (Fig. 3E–H). This finding is in agreement with previous observations derived from transmission immunoelectron microscopy images in which the s36 protein could not be detected in the ICL layer^18^,^21^. The same studies also reported immunolocalization of s36 chorionic protein in the vitelline membrane, a layer that has already been formed during pre-choriogenic developmental stages. However, our s36-targeted follicles present an electron-dense vitelline membrane with no obvious structural deformities (Fig. 3C,D). Furthermore, the vitelline membrane of s36-depleted follicles does not seem to carry any functional defect, such as loss of permeability control, as demonstrated by the inability of neutral-red reagent to penetrate the s36-targeted or control de-chorionated eggs (Fig. 4A,B). This suggests a non-essential role of s36 protein in vitelline-membrane morphogenesis and function.

Altogether, our data unearth the fundamental contribution of s36 protein to the development of chorion’s regional specialization (Fig. 2) and radial complexity (Fig. 3). Moreover, they indicate the inability of other chorionic proteins to successfully compensate for the lack of s36 during biogenesis of fly chorion.

### *Drosophila melanogaster* eggshell of s36-targeted -laid- follicles suffers from surface deformity and increased fragility: an AFM-mediated quantification of its mechanical strength

**Figure 5:**
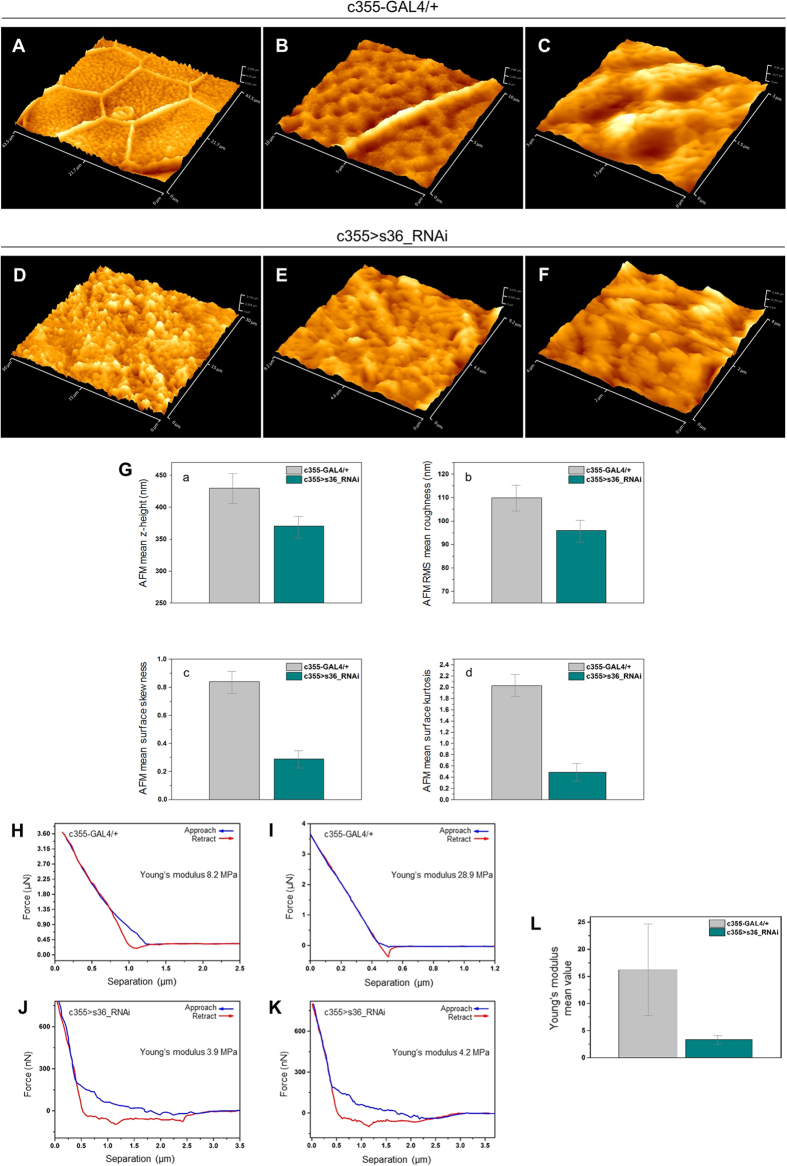
AFM-mediated imaging, and quantification of eggshell’s surface deformity and fragility in s36-targeted ovarian follicles. Atomic force microscopy imaging of control (c355-GAL4/+) (**A**–**C**) and s36-depleted (c355 > s36_RNAi) (**D**–**F**) laid follicles. (**A**) Representative follicular imprint in a hexagonal form on control follicle’s surface. (**B**) Typical side (~1 μm width and ~400 nm height) of a follicular imprint that specifies control follicles. (**C**) Canonically distributed “hill and valley” structures within a representative hexagonal area of control follicle. The s36-depleted follicles lack the characteristic follicular imprints (**D**,**E**) and “hill-valley” typical assemblies (**F**). (Ga–Gd) Mean values of surface -geometrical- parameters, such as z-height (Ga), RMS roughness (Gb), skewness (Gc) and kurtosis (Gd) of control and s36-depleted follicles. (**H–K**) Lower (**H**,**J**) and upper (**I**,**K**) boundaries of force-distance curves of control (**H**,**I**) and s36-downregulated (**J**,**K**) -laid- follicles. (**L**) Young’s modulus average values of control and s36-targeted follicles.

AFM in contrast to SEM is operated in ambient environment and provides a greater level of detail for anatomically smooth surfaces^22^. Therefore, AFM imaging was herein employed for a high-resolution quantification of eggshell topography and mechanical strength of control and s36-targeted eggs. Eggshell-surface topography of an approximately 45 × 45 μm^2^ area of a representative control follicle was defined by arrays of typical hexagonal formations (Fig. 5A) being created by chorionic material secreted from the above follicle cells. As illustrated in higher-magnification images, the hexagon sides vary in length but consist of approximately 1 μm in width and 400 nm in height solenoid-like structures, so-called follicular imprints or boundaries (Fig. 5B and data not shown). Within each hexagon area, spherically organized structures of approximately 800 nm in average size are self-assembled in a “hill-valley” configuration (Fig. 5C) and rather canonical distribution (Fig. 5B,C). This hexagonal-type architecture is relatively more compatible with the flattest part of oval surfaces, as they bear minimum strains among adjacent boundaries in agreement with the major evolutionary principle of optimum viability. On the contrary, pentagonal structures appear more frequently on the curved parts of eggshell’s surface (Fig. 2E and data not shown). Interestingly, AFM surface-topography images of s36-targeted follicles revealed the absence of follicular imprints (Fig. 5D,E) and typical “hill-valley” assemblies (Fig. 5E,F). The disorganized surface is mainly composed of amorphous, and diverse-size and -shape structures that are randomly arranged. Furthermore, four surface parameters were quantified and their mean values are herein shown in Fig. 5G. In all cases of z-height (Fig. 5Ga), RMS roughness (Fig. 5Gb), skewness (Fig. 5Gc) and kurtosis (Fig. 5Gd) examined, the s36-depleted follicles were presented with notably reduced values compared to control ones.

Force spectroscopy, which has no SEM counterpart, represents an approach-retract mode of AFM that can be employed to assess cell-surface compliance and elasticity^23^. Surface AFM imaging with different spring constant tips revealed that s36-downregulated follicles were far more brittle compared to control ones. The elastic modulus was ranged between 8.2 and 28.9 MPa for control (Fig. 5H,I), and 3.9 and 4.2 MPa for s36-depleted (Fig. 5J,K) follicles. Mean values of Young’s modulus for control and s36-targeted follicles were calculated as 16.2 ± 8.3 and 3.3 ± 0.7 MPa, respectively (Fig. 5L). This indicates that lack of s36 renders fly follicles approximately five times more fragile to applied mechanical stress as compared to control ones. Altogether, the obtained AFM quantitative data could be used as reference for future comparisons with phenotypes of other eggshell-protein gene disruptions.

### Follicle cell-specific depletion of s36 chorionic protein results in irregular peroxidase-activity patterning and fly sterility

**Figure 6:**
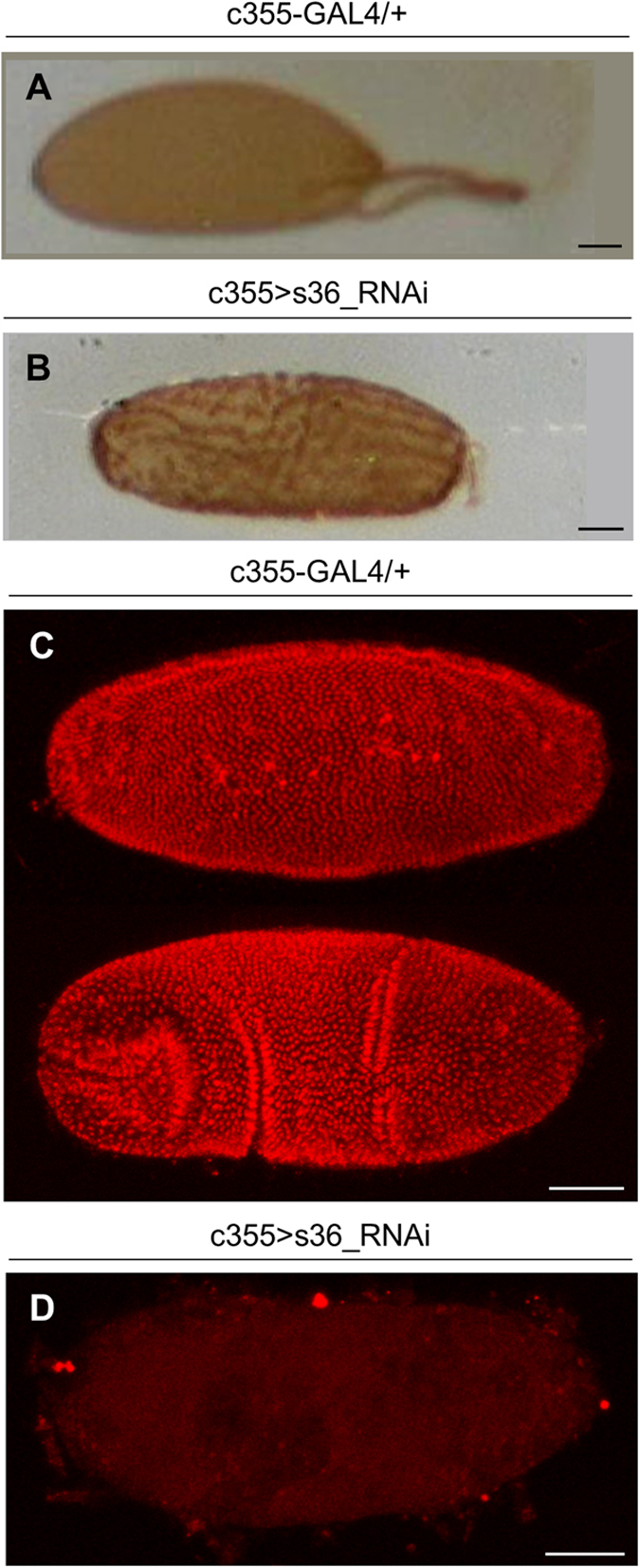
Depletion of s36 protein in the follicle-cell cluster of *Drosophila melanogaster* ovary is associated with perturbed patterning of chorionic peroxidase activity and absence of post-zygotic mitotic divisions in early embryogenesis. (**A**,**B**) Light microscopy images of a DAB-based assay for peroxidase activity in control (c355-GAL4/+) (**A**) and s36-downregulated (c355 > s36_RNAi) (**B**) follicles. (**C**,**D**) Confocal laser scanning microscopy images, after a PI nuclear-staining process, of two control (de-chorionated and de-vitellinized) embryos at different developmental stages (**C**) and a representative s36-downregulated (de-chorionated and de-vitellinized) laid follicle that appeared unable to proceed to early embryogenesis as dictated by the absence of PI-positive post-zygotic nuclei (**D**). Scale Bars: 50 μm.

The increased eggshell’s fragility observed in s36-downregulated follicles (Fig. 5) may likely derive from perturbation of endochorionic-hardening process. *Drosophila* eggshell is stabilized and becomes insoluble during stage 14 via regulated activation of an endogenous peroxidase that is able to cross-link two or three tyrosine residues of eggshell’s proteins rendering them largely insoluble^24^. Peroxidase enzyme is secreted by the follicle-cell cluster and considered as a structural component of endochorion and ICL^7^. Its activation occurs at the end of choriogenesis by H_2_O_2_ secreted by follicle cells^25^. In order to correlate eggshell’s fragility with hardening-process aberration of s36-depleted endochorion, we applied an *in situ* histochemical assay according to which a peroxidase-catalyzed oxidation of DAB by H_2_O_2_ leads to a brown coloration. An irregular patterning (e.g. spotty- and filamentous-like areas) of chorionic peroxidase activity was recognized in s36-targeted follicles (Fig. 6B) as compared to the typical respective one in control follicles (Fig. 6A). These data indicate that the s36 lack-induced collapse of endochorionic primary scaffold likely causes incomplete formation of di- and tri-tyrosine bonds, thus compromising eggshell’s hardening capacity.

Next, in an effort to examine whether the herein revealed chorionic disintegration of s36-downregulated follicles could disturb fly’s fecundity, a hatchability assay was performed. In contrast to control flies, the s36-targeted ones were presented with an eliminated eclosion efficiency (data not shown). Defective hatching may derive from failure in egg fertilization or embryogenesis. Interestingly, no post-zygotic mitotic nucleus could be observed in the s36-targeted laid follicles (Fig. 6D) in contrast to control ones that all developed to embryos (Fig. 6C). Therefore, it seems that the absence of s36 likely renders ovarian follicles incapable to be fertilized by the sperm.

### Targeting the s36 chorionic protein in regionally specialized follicle-cell subpopulations does not disturb micropylar and polar integrity of ovarian eggshell

**Figure 7:**
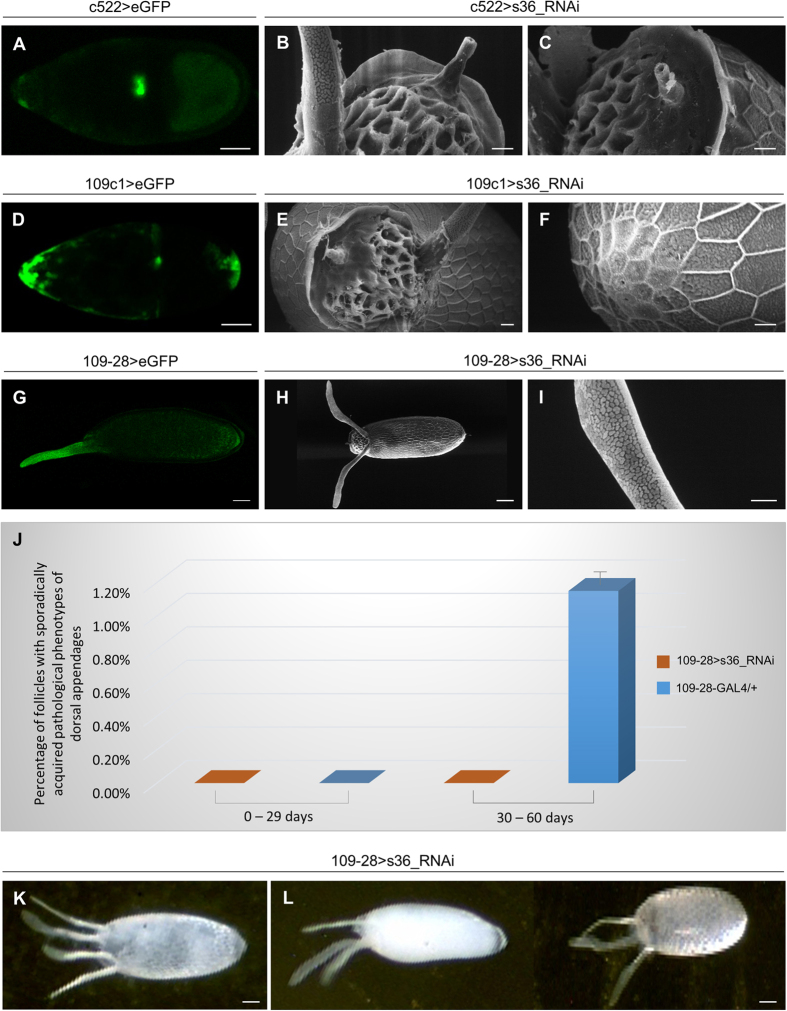
Lack of s36 chorionic protein from regionally specialized follicle-cell subpopulations does not harm micropylar or polar integrity, but causes an age-dependent sporadic derailment of dorsal-appendages’ patterning. (**A**,**D**) Confocal laser scanning microscopy (CLSM) images of representative stage-10 control follicles specifically expressing the eGFP protein either in the border-cell cluster (c522 > eGFP) (**A**) or in the (anterior and posterior) polar-cell clusters (109c1 > eGFP) (**D**). (**B**,**C**) Scanning electron microscopy (SEM) images of follicles being specifically targeted for the s36 protein in border cells (c522 > s36_RNAi). (**E**,**F**) SEM images of follicles being specifically targeted for the s36 protein in (anterior and posterior) polar cells (109c1 > s36_RNAi). (**E**) Anterior end. (**F**) Posterior end. (**G**) CLSM image of a representative stage-14 control follicle specifically expressing the eGFP protein in follicle cells of dorsal appendages (109-28 > eGFP). (**H**,**I**) SEM images of follicles being specifically targeted for the s36 protein in follicle cells of dorsal appendages (109-28 > s36_RNAi). (**J**) Bar-chart presenting the percentage (%) of follicles with a sporadically acquired and fly age-driven (0–29 and 30–60 days) pathology of dorsal appendages being produced by 109-28 > s36_RNAi but not 109-28-GAL4/+ (control) fly strains. (**K**,**L**) Light microscopy images of 109-28 > s36_RNAi follicles being sporadically generated by aged flies. Dysmorphic follicles are characterized by the development of four (**K**) or three (**L**), instead of two (e.g. Fig. 7H), dorsal appendages. Scale Bars: 50 μm in **A,D,G,H,K,L**; 10 μm in **B,C,E,F,I**.

Through RNA *in situ* hybridization it has been previously reported that *s36* chorionic gene is first activated within a small dorsal subpopulation of stage 10B follicle cells, while at later stages it is expressed in virtually all follicle cells. Furthermore, apart from a small group of follicle cells located at the tip of micropyle, all the other follicle cells responsible for micropyle formation express the s36 chorionic protein^26^. Hence, in an effort to determine the potential role of s36 in micropyle morphogenesis, we proceeded to the RNAi-mediated downregulation of s36 protein in border cells, through utilization of the c522-GAL4 driver (Fig. 7A), or in polar cells (at both the anterior and posterior poles), with employment of the 109c1-GAL4 driver (Fig. 7D). The specificity of each driver was assessed through eGFP-directed CLSM imaging. Strong GFP expression was detected throughout all the developmental stages in which s36 chorionic protein is expressed (Fig. 7A,D and data not shown). It is well-known that at early-stage 9 anterior polar cells recruit 4-6 adjacent follicle cells in order them to become border cells and to migrate posteriorly. By stage 10, these border cells reach the anterior end of the oocyte where they participate in the formation of micropylar pore^7^,^27^. Micropylar apparatus of follicles that have been targeted for s36 chorionic protein in the border-cell cluster (Fig. 7B,C) or polar-cell clusters (Fig. 7E) are presented with unaffected morphology and physiological porthole entry. Moreover, these transgenic flies demonstrate typical eclosion and fecundity levels (Fig. S2A–D).

Polar cells also provide cues for embryonic and eggshell axial patterning, and orchestrate follicle-cell fates throughout oogenesis^8^. Flies targeted for the s36 chorionic protein in polar cells proved to bear physiological follicles (Fig. 7E,F, and data not shown). It seems that the targeted downregulation of s36 chorionic protein in border or polar cells is not a sufficient condition to cause any structural or functional defect. Alternatively, it is possible that even limited amounts of s36 chorionic protein secreted from the adjacent unaffected follicle cells are likely adequate to preserve eggshell’s architectural shape. Nevertheless, we cannot rule out the possibility that the GAL4 drivers used in this study do not confer strong GAL4 expression and therefore sufficient gene downregulation.

### Downregulation of s36 chorionic protein in the follicle-cell cluster of dorsal appendages compels them to sporadically undergo deformed patterning and architectural dysmorphia in elderly flies

**Figure 8:**
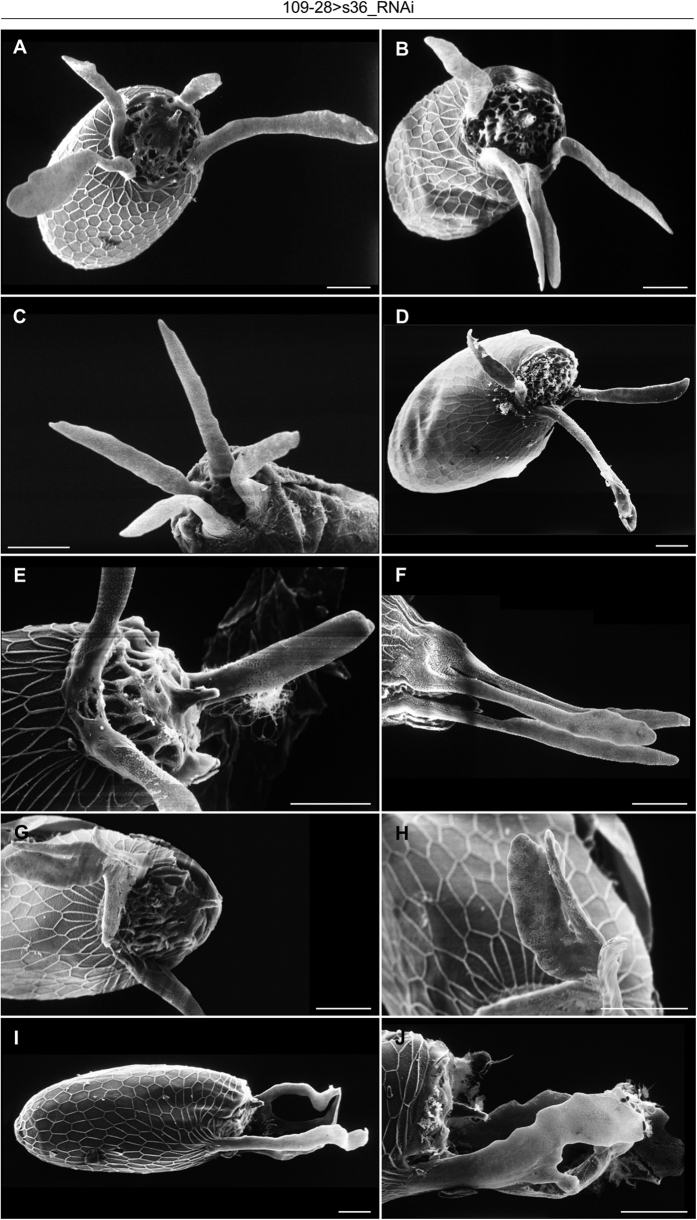
Targeted depletion of s36 chorionic protein in the follicle-cell cluster of dorsal appendages sporadically induces a broad spectrum of follicular-pathology phenotypes in elderly flies. (**A**–**J**) Scanning electron microscopy images of 109-28 > s36_RNAi transgenic -dysmorphic- follicles with pleiotropic dorsal-appendages’ pathology being sporadically produced by aged flies. (**A**,**B**) Four normally-sized dorsal appendages with almost typical paddle-shaped tips. (**C**) Appendages with abnormal tips. (**D**,**F**) Three almost typical dorsal appendages. (**E**) Two typically-shaped and -sized appendages together with an extra short and stubby one. (**G**,**H**) Dorsal appendages with bulky bifurcated tips (H: higher magnification of G). (**I**,**J**) Fused and antler-shaped dorsal appendages. Scale Bars: 50 μm.

Reduced expression of s36 chorionic protein specifically in follicle cells of dorsal appendages via utilization of the 109-28-GAL4 driver (Fig. 7G and data not shown) did not seem to detectably affect the physiological structure of dorsal appendages (Fig. 7H,I) or typical eclosion and fecundity profiles (Fig. S2E,F). However, aged (30–60 days old) flies proved to contain in their ovaries a low percentage (Fig. 7J) of follicles carrying derailed patterning and/or dysmorphic architecture of dorsal appendages (Figs 7K,L and 8). A broad spectrum of pathological phenotypes ranging from follicles with three or four, rather typical, dorsal appendages (Figs 7K,L and 8A,B,D,F) to follicles with extremely deformed and fused dorsal appendages (Fig. 8G–J) was observed. Surface analysis through SEM technology recognized follicles bearing (a) three (Fig. 8D,F) or four (Fig. 8A,B) regularly sized dorsal appendages with almost typical paddle-shaped tips, (b) two or three typically shaped and sized appendages, and an extra short and stubby one (Fig. 8E)(c) appendages with abnormal tips (Fig. 8C) and (d) other pleiotropic phenotypes of dysmorphic features (Fig. 8G–J). A fraction of these follicles was typified by dorsal appendages with bulky bifurcated tips (Fig. 8G,H) or by four dorsal appendages of an antler-like shape (Fig. 8I,J). This novel phenotype indicates that s36 chorionic protein can likely protect signaling integrity of follicle cells responsible for dorsal appendages’ biogenesis from age-induced stress.

## Discussion

Besides its indispensable role in *Drosophila* oogenesis, chorion serves as an *in vivo* model system for the investigation of mechanisms controlling multi-protein structural assembly and cell lineage-specific gene expression. Hence, an improved insight into the importance of chorion’s components in ovarian-follicle integrity is required. A previous study based on a mutation being cytogenetically mapped to region 7E10-8A4 of the X fly chromosome has described eggshells with structural abnormalities in the endochorionic layer^7^,^28^. However, this 16-band broad chromosomal region contains 40 genes, five of which (*Cp7Fa*, *Cp7Fb*, *Cp7Fc*, *s36* and *s38*) are classified as typical chorionic genes^29^. To the same direction, *Cp36*^*dec2-1*^ was previously suggested to behave as a strong hypomorphic mutation of the *s36* chorionic gene^30^. Nevertheless, this mutation has been cytogenetically mapped to the region 7E10-8A5^30^ that contains 41 genes (including the 5 chorionic ones)^29^. Follicles from *Cp36*^*dec2-1*^ mutants were shorter and rounder with short dorsal appendages, fragile chorion (that is stripped away when the egg is laid) and defects in vitelline membrane^31^,^32^. Furthermore, the *ocelliless* mutation (that disrupts chorion-gene amplification process and reduces the expression of both s36 and s38 proteins^33^) is characterized by a thin, though organized, endochorion and reduced number of beta-yolk spheres^34^,^35^. Therefore, since there is no convincing evidence that the phenotypes derived from the previous mutations are directly attributed to the s36-specific impairment, we have herein aimed to illuminate via employment of an RNAi-based technology the *in vivo* role of s36 chorionic protein in *Drosophila* eggshell’s biogenesis and architecture.

Surface analysis revealed that s36-depleted follicles were presented with a thin and fragile chorion that lacked typical honeycomb surface structures, and also bore severely malformed and rudimentary dorsal appendages. Ultrastructural follicle’s examination indicated that s36 chorionic protein plays a fundamental role in the architectural formation of a structural scaffold. Integration of s36 in this primary pre-endochorionic layer is likely required in order for the other chorionic proteins to be properly recruited and bound onto it for the successful molding of fly’s chorion body. Lack of s36 causes abundant accumulation of amorphous endochorionic material that is randomly aggregated in irregular clumps and is therefore prohibited from being organized in its typical tripartite structure (Fig. 3).

Our findings unearth the critical contribution of s36 protein in patterning establishment of chorion’s regional specialization (Fig. 2) and radial complexity (Fig. 3). Moreover, they also indicate the inability of other chorionic family members and especially the s38 one (whose cognate X-linked gene is amplified and developmentally regulated in a similar to *s36* manner^7^) to compensate for the lack of s36 synthesis from ovary’s follicle cell-cluster. Interestingly, the variety of dysmorphic phenotypes recognized in s36-targeted follicles (Fig. 3) suggests that s36 can operate as regulator of chorion-assembly developmental randomness likely being engaged at the beginning of choriogenesis.

AFM surface analysis evidenced the principal role of s36 protein in the maintenance of chorion’s mechanical strength. The s36-downregulated follicles presented an approximately five-times increased fragility as compared to control ones. Besides the mechanical, other geometrical traits (e.g. surface roughness, skewness and kurtosis) were also perturbed, thus revealing the s36 protein’s contribution to chorion’s architectural geometry (Fig. 5). Increased fragility seems to be associated with defective hardening of the s36-depleted eggshells. This may be due to inefficient formation of di-tyrosine and tri-tyrosine covalent bonds among different chorionic proteins, as indicated by the irregular patterning of peroxidase activity in s36-downregulated follicles (Fig. 6). A collapsed endochorionic scaffold that lacks s36 cannot support the successful cross-linking of chorionic components, resulting in biogenesis of a mechanically fragile eggshell. Perturbation of protein cross-linking mechanism may disable chorion from performing its physiological functions during embryonic development^4^,^7^.

Laid s36-targeted follicles proved unable to hatch likely due to defects in vitelline-membrane and/or micropyle integrity. *Cp36*^*dec2-1*^ mutant follicles were characterized by vitelline-envelope abnormalities^31^ and vitelline-membrane cross-linking compromised capacity. Specifically, *Cp36*^*dec2-1*^ de-chorionated follicles demonstrated significant uptake of neutral red, a dye that is normally unable to cross the vitelline-membrane barrier^32^. In addition, a role of s36 protein in vitelline-membrane biogenesis was further supported by the immunolocalization of s36 chorionic protein in vitelline-membrane layers of *D. melanogaster*^18^ and *D. virilis*^21^. However, according to our TEM images (Fig. 3) and neutral-red assays (Fig. 4), the s36-targeted follicles were presented with structurally unharmed and functionally impermeable vitelline membrane. Moreover, in contrast to *Cp36*^*dec2-1*^ follicles that burst upon de-chorionation^31^,^32^, the s36-depleted ones remain unaffected (Fig. 4). The aforementioned data indicate the non-essential role of s36 chorionic protein in architectural formation and physiological function of vitelline membrane. *Cp36*^*dec2-1*^ follicles, besides *s36*, are missing numerous other genes^29^,^30^, one or more of which could be associated with vitelline-membrane’s pathological phenotype observed^32^. As previously proposed, an amount of s36 protein likely makes its way to the vitelline-membrane layer where it becomes transiently sequestered. During late choriogenesis, the sequestered molecules are released from vitelline-membrane reservoir and become available for immediate incorporation into the growing chorionic structure^7^,^18^.

One possible cause for the observed sterility of s36-targeted flies may be a defective micropyle. At the end of micropylar biogenesis the two cytoplasmic projections (full of cytoskeletal elements) that were secreted from border cells to function as a mold for the formation of micropylar canal are withdrawn leaving endochorion as the only supportive structure to keep the pore open^27^,^36^. It is likely that in s36-depleted follicles the lack of a solid endochorionic scaffold (together with the altered orientation of exochorionic fibers^36^ and the presence of a thinner ICL layer^36^) can cause a collapse of chorionic material inside the micropylar pore resulting in its structural occlusion (Fig. 2). In accordance, the inability of s36-targeted laid eggs to proceed to early embryogenesis (Fig. 6) indicates a problem in sperm entry. This supports the notion that a defective micropyle in s36-depleted follicles leads flies to infertility. Nevertheless, despite that the laid (Fig. 2B) and de-chorionated (Fig. 4B) s36-depleted eggs do not appear to suffer from major dehydration, we cannot rule out the possibility that collapsed chorion promotes a low-scale follicle’s desiccation which ultimately leads to fly’s infertility.

Dorsal appendages bear a dorsal surface covered with porous plaques similar to pits of the endochorionic floor and a ventral surface much alike to the endochorionic-roof network^5^,^6^. This endochorionic-type structure justifies the formation of short, thin and collapsed dorsal appendages in the s36-targeted follicles (Fig. 2). Anterodorsal patterning and development of dorsal appendages require the convergence of two signaling pathways; the Gurken (Grk)- and Decapentaplegic (DPP)-dependent ones^37^,^38^. Grk- and DPP-driven pathways overlap on two distinct follicle-cell subpopulations that are the progenitor cells of dorsal appendages and are characterized by *Broad-Complex* (*BR-C*) gene expression^1^,^12^,^37^. DPP loss-of-function mutations generate short and -often- paddle-less appendages, whereas DPP over-expression creates multiple and -frequently- antler-shaped dorsal appendages^39^,^40^,^41^. In addition, changes in distribution and amount of Grk protein can lead to development of follicles with zero to four dorsal appendages^42^,^43^. Variations in signaling emanated from Torpedo, the Grk receptor^37^,^39^, cause alterations in the number and concavity of BR-C patches. Partial loss-of-function mutants, in which the size and number of dorsal appendages were modified when the size and number of BR-C-expressing cells were changed, demonstrate that BR-C-protein activities are essential for dorsal appendages’ morphogenesis^39^. It is this differential regulation of BR-C patches that controls the distinct number of dorsal appendages between *D. virilis* (four) and *D. melanogaster* (two)^43^,^44^. The phenotypic similarities among the s36-depleted, DPP- and Grk-mutant follicles suggest that the s36 chorionic protein may synergize with the two signaling pathways to specify the BR-C-cell complex. Alternatively, s36 could operate as modulator or mediator of DPP and/or Grk signaling. Interestingly, the precise *s36* gene-expression patterning (as dictated by its proximal regulatory region) has been shown to depend on Torpedo signaling^45^. Altogether, the sporadic pathology of dorsal appendages observed in follicles being targeted for s36 chorionic protein in the follicle-cell cluster of dorsal appendages (Fig. 8) implies that s36 may regulate, under conditions of age-induced stress, the correct localization, number and concavity of BR-C patches. As previously reported, perturbations in concavity could lead to mechanical instabilities that further subdivide the BR-C cells into smaller domains, hence promoting the formation of extra dorsal appendages^44^.

Conclusively, s36 proves to operate as a cardinal architectural protein which controls the assembly of a structural scaffold that supports chorion’s regional specialization, radial complexity and mechanical strength during *Drosophila* oogenesis. Therefore, chorion’s biogenesis can be exploited for illumination of mechanisms governing the self-assembly processes of multi-protein systems and the follicular pathology-mediated sterility.

## Additional Information

**How to cite this article**: Velentzas, A. D. *et al*. Targeted Downregulation of s36 Protein Unearths its Cardinal Role in Chorion Biogenesis and Architecture during *Drosophila melanogaster* Oogenesis. *Sci. Rep.* **6**, 35511; doi: 10.1038/srep35511 (2016).

## Supplementary Material

Supplementary Information

Supplementary Table S2
