# Supplementary material for: Targeted Downregulation of s36 Protein Unearths its Cardinal Role in Chorion Biogenesis and Architecture during Drosophila melanogaster Oogenesis
Source: Sci Rep. 2016 Oct 18;6:35511. doi: 10.1038/srep35511 (PMC5067561; doi:10.1038/srep35511)
Supplement: Supplementary Information [file srep35511-s1.pdf]

Targeted Downregulation of s36 Protein Unearths its Cardinal Role in Chorion Biogenesis and Architecture during *Drosophila melanogaster* Oogenesis

Athanassios D. Velentzas[1], Panagiotis D. Velentzas[1,2], Niki E. Sagioglou[1], Eumorphia G. Konstantakou[1,3], Athanasios K. Anagnostopoulos[4], Maria M. Tsioka[1], Vassiliki E. Mpakou[1], Zoe Kollia[5], Christos Consoulas[6], Lukas H. Margaritis[1], Issidora S. Papassideri[1], George Th. Tsangaris[4], Evangelia Sarantopoulou[5], Alkiviadis-Constantinos Cefalas[5] and Dimitrios J. Stravopodis[1,*]

[1]Section of Cell Biology and Biophysics, Department of Biology, School of Science, National and Kapodistrian University of Athens (NKUA), Athens, Greece

[2]Present Address: Department of Cancer Biology, Medical School, University of Massachusetts, Worcester, Massachusetts (MA), USA

[3]Present Address: Department of Hematology and Medical Oncology, Winship Cancer Institute of Emory, Emory University School of Medicine, Atlanta, Georgia (GA), USA

[4]Proteomics Core Facility, Systems Biology Center, Biomedical Research Foundation of the Academy of Athens (BRFAA), Athens, Greece

[5]Theoretical and Physical Chemistry Institute, National Hellenic Research Foundation (NHRF), Athens, Greece

[6]Laboratory of Experimental Physiology, Medical School, National and Kapodistrian University of Athens (NKUA), Athens, Greece

[*]**Corresponding Author**:

Dimitrios J. Stravopodis, *BSc*, *MSc*, *PhD*, Assistant Professor, Section of Cell Biology and Biophysics, Department of Biology, School of Science, National and Kapodistrian University of Athens (NKUA), Panepistimiopolis, Zografou 157 84, Athens, Greece (GR). Tel.: +30 210 727 4105, Fax: +30 210 727 4742, E-mail: dstravop@biol.uoa.gr, URL: http://www.biol.uoa.gr

Running Title: Indispensable Contribution of s36 Protein to Chorion Morphogenesis

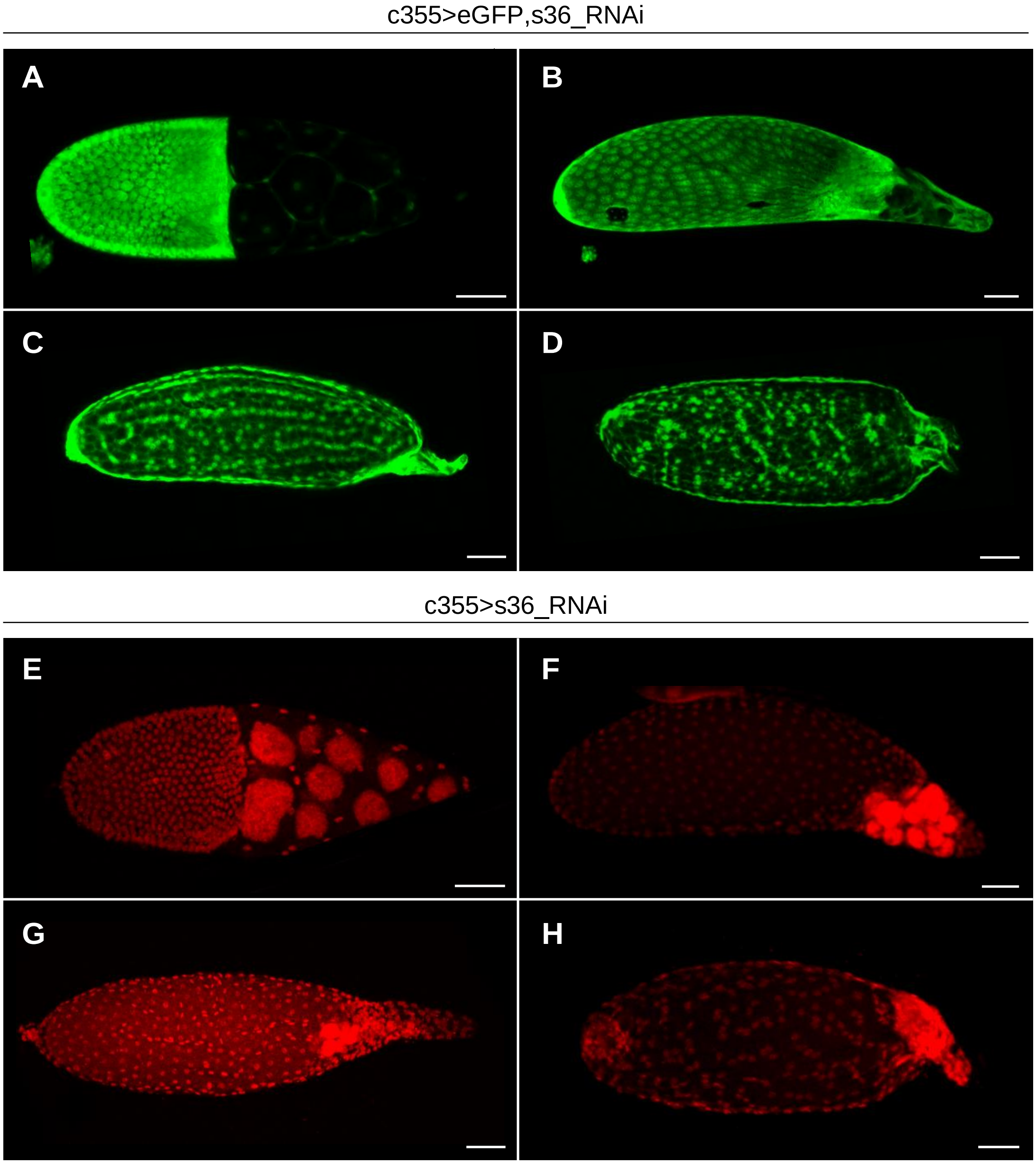
c355>eGFP,s36_RNAi
A
B
C
D
c355>s36_RNAi
E
F
G
H

Supplementary Figure S1

The developmental courses of follicle- and nurse-cell clusters remain unaffected in the absence of s36 chorionic protein. (A-D) Confocal laser scanning microscopy (CLSM) images of eGFP-protein expression profiling in the s36-depleted follicle-cell cluster of triple transgenic (c355>eGFP,s36_RNAi) ovarian follicles at developmental stage 10 (A), 13 (B) and 14 (C and D). (E-H) CLSM imaging of PI nuclear staining of follicle- and nurse-cell compartments in the s36-targeted follicles (c355>s36_RNAi) at developmental stage 10 (E), 12 (F), 13 (G) and 14 (H). Scale Bars: 50 μm.

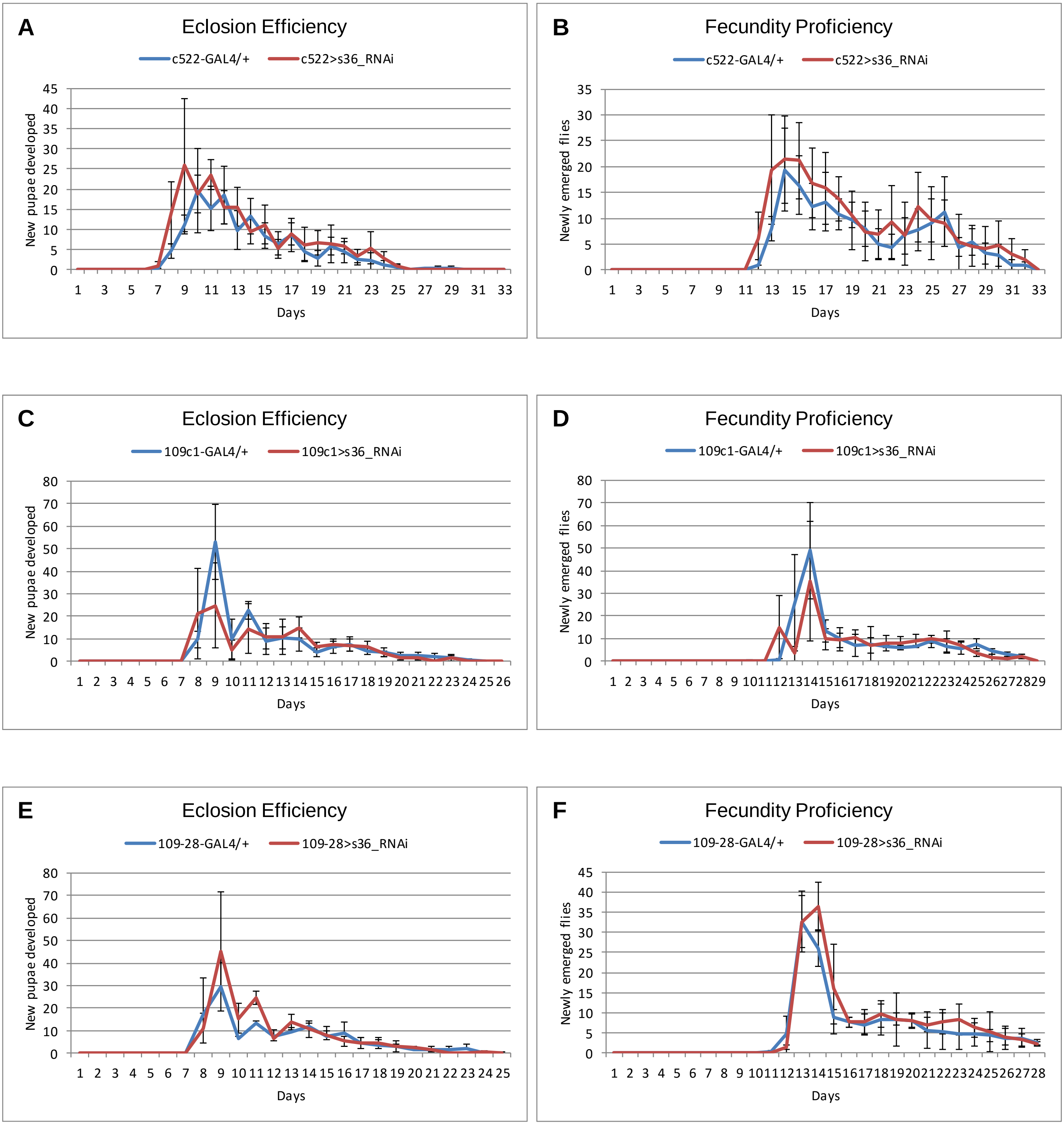
A
Eclosion Efficiency
c522-GAL4/+
c522>s36_RNAi
New pupae developed
Days
B
Fecundity Proficiency
c522-GAL4/+
c522>s36_RNAi
Newly emerged flies
Days
C
Eclosion Efficiency
109c1-GAL4/+
109c1>s36_RNAi
New pupae developed
Days
D
Fecundity Proficiency
109c1-GAL4/+
109c1>s36_RNAi
Newly emerged flies
Days
E
Eclosion Efficiency
109-28-GAL4/+
109-28>s36_RNAi
New pupae developed
Days
F
Fecundity Proficiency
109-28-GAL4/+
109-28>s36_RNAi
Newly emerged flies
Days

Supplementary Figure S2

Genetic targeting of s36 chorionic protein in regionally specialized follicle-cell subpopulations does not harm typical eclosion capacity and fecundity integrity. Eclosion-efficiency (A, C and E) and fecundity-proficiency (B, D and F) graphs of flies being targeted for the s36 chorionic protein specifically in follicular border cells (c522>s36_RNAi) (A and B), (anterior and posterior) polar cells (109c1>s36_RNAi) (C and D), or follicle cells of dorsal appendages (109-28>s36_RNAi) (E and F). Note the significant similarities with their respective control (c522-GAL4/+, 109c1-GAL4/+ and 109-28-GAL4/+) fly strains.

Gene Name	Primer Sequence	Molecular Size (bp) of PCR Product	Ta
Chorion protein 36 (CG1478)	F: 5′-CAA CTC GGT CTC TGG TTT GG-3′<br>R: 5′-CTG TGA GGC GAT CTG ATT GG -3′	195	57ºC
Chorion protein 38 (CG11213)	F: 5′-CGA GAT CGA CCT ACA TTT G-3′<br>R: 5′-ATT CGA GAG CAC CAG CAC C-3′	213	53ºC
Chorion protein 18 (CG6517)	F: 5′-GCC AAT GTG GGA TCT CAG TA-3′<br>R: 5′-GCT GTG TCC GTA CTC CTG GT-3′	195	55ºC
Chorion protein 16 (CG6533)	F: 5′-AAG CTG GAC GGT GCT GAC T-3′<br>R: 5′-GGT CTT CAG GTA CGC ATT C-3′	201	53ºC
Chorion protein 19 (CG6524)	F: 5′-GCT ACA ACG GTG GCA ACT A-3′<br>R: 5′-CCA TAC TGG TTC TCT GGG A-3′	196	53ºC
Chorion protein 15 (CG6519)	F: 5′-AGG TTA TGG TGG TGG CTA CG-3′<br>R: 5′-CTT GTA GGT TCC GGG ATT CA-3′	190	55ºC

Supplementary Table S1

Gene name, oligonucleotide primer sequence (F: forward; R: reverse), molecular size (bp) and annealing temperature (Ta) of the amplified PCR products for the herein examined genes

Supplementary Table S2 (a separate Microsoft Excel format file)

Protein library (N = 2,131 members) of s36-depleted fly ovaries (c355>s36_RNAi) indicating for each identified protein its: (a) UniProt accession number, (b) name and description together with fundamental features of LC-MS/MS proteomics analysis, such as (c) (Mascot) score, (d) sequence coverage, (e) number of unique peptides (n = 17,772 tryptic fragments), (f) number of amino-acid residues (AAs), (g) molecular weight (MW) in kDa and (h) (calculated) isoelectric point (pI)
